# VPS34 complexes from a structural perspective

**DOI:** 10.1194/jlr.R089490

**Published:** 2018-11-05

**Authors:** Yohei Ohashi, Shirley Tremel, Roger L. Williams

**Affiliations:** MRC Laboratory of Molecular Biology, Cambridge CB2 0QH, United Kingdom

**Keywords:** vacuolar protein sorting 34, X-ray crystallography, cryo-electron microscopy, hydrogen-deuterium exchange mass-spectrometry, lipid

## Abstract

VPS34 phosphorylates phosphatidylinositol to produce PtdIns3P and is the progenitor of the phosphoinositide 3-kinase (PI3K) family. VPS34 has a simpler domain organization than class I PI3Ks, which belies the complexity of its quaternary organization, with the enzyme always functioning within larger assemblies. PtdIns3P recruits specific recognition modules that are common in protein-sorting pathways, such as autophagy and endocytic sorting. It is best characterized in two heterotetramers, complexes I and II. Complex I is composed of VPS34, VPS15, Beclin 1, and autophagy-related gene (ATG)14L, whereas complex II replaces ATG14L with UVRAG. Because VPS34 can form a component of several distinct complexes, it enables independent regulation of various pathways that are controlled by PtdIns3P. Complexes I and II are critical for early events in autophagy and endocytic sorting, respectively. Autophagy has a complex association with cancer. In early stages, it inhibits tumorigenesis, but in later stages, it acts as a survival factor for tumors. Recently, various disease-associated somatic mutations were found in genes encoding complex I and II subunits. Lipid kinase activities of the complexes are also influenced by posttranslational modifications (PTMs). Mapping PTMs and somatic mutations on three-dimensional models of the complexes suggests mechanisms for how these affect VPS34 activity.

## VPS34: THE FOUNDING MEMBER OF THE PI3K FAMILY

**Fig. 1.**
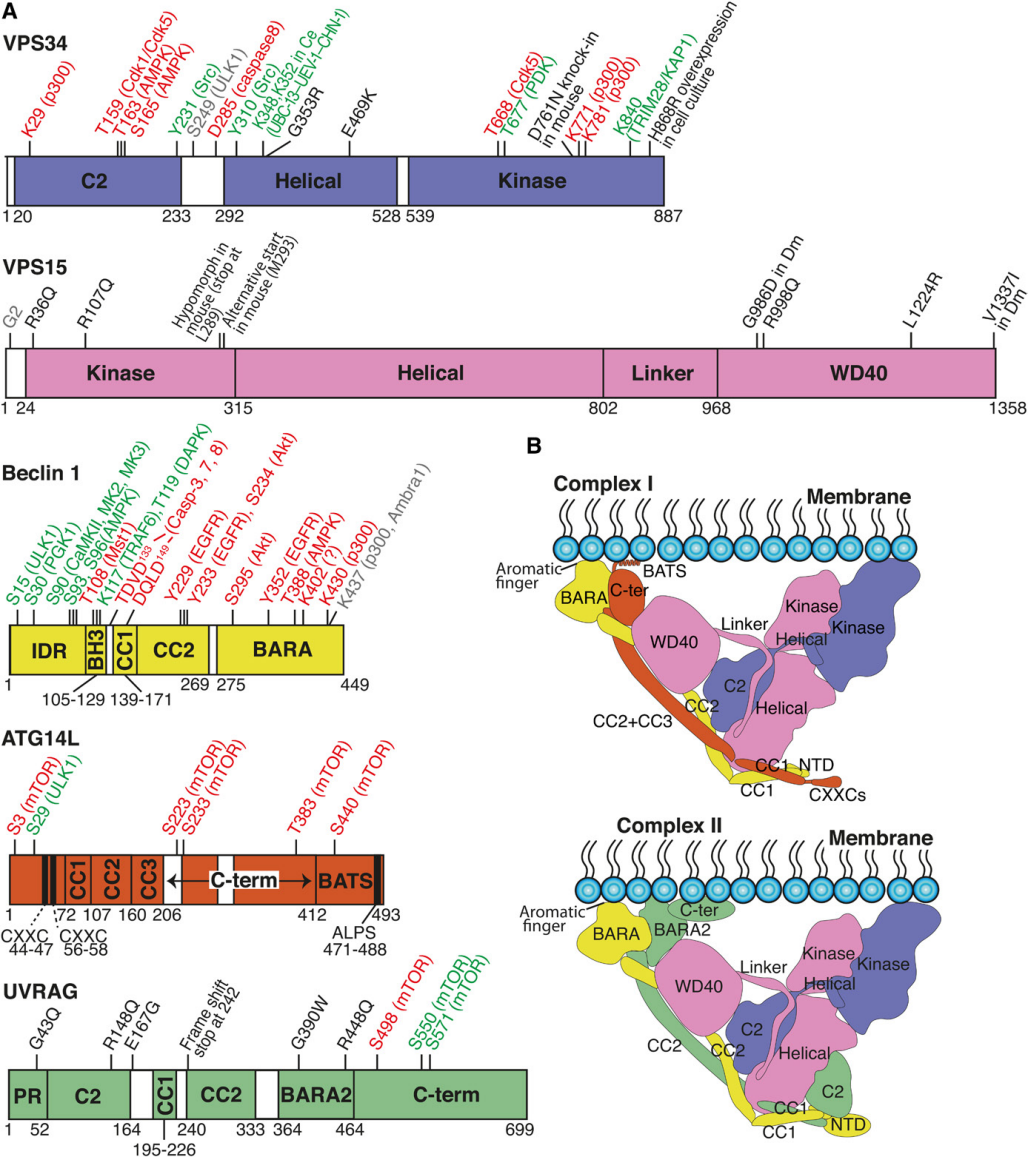
Structures of complex I and complex II. A: Schematic representations for the subunits of the class III PI3K complexes. Posttranslational modifcations (PTMs) and somatic mutations are indicated on the upper side of each subunit. Unless otherwise noted, all residue numbers are for the human sequences. Ce, *C. elegans*; Dm, *D. melanogaster*. Red, inhibiting; green, activating; gray, no effect or either inhibiting or activating; black, somatic mutations. B: Schematic structural models of complex I (top) and complex II (bottom). Because structural information on the CXXC, C-ter, and BATS regions of ATG14L, and the NTD and C-ter of UVRAG is not available, the boundaries of these domains are speculative.

The phosphoinositide 3-kinases (PI3Ks) are a family of intracellular lipid kinases that are unique to eukaryotic cells. The enzymes all phosphorylate the 3-OH of inositol lipids, but they can be grouped into three classes based on their domain organization. The class I PI3Ks evolved in metazoa and use phosphatidylinositol 4,5-bisphosphate [PtdIns(4,5)P2] to produce PtdIns(3,4,5)P3, which has a role as a membrane-resident second messenger. The class II PI3Ks have a C-terminal PX-C2 domain extension to the class I-like core, and they have an N-terminal region unrelated to the adaptor-binding domains of the class I enzymes. While there are four class I and three class II PI3Ks in mammalian cells, there is only one class III enzyme, VPS34, and given that it is present in all eukaryotes, it represents the primordial PI3K. VPS34 has a three-domain architecture consisting of an N-terminal C2 domain, a helical domain, and a C-terminal kinase domain that is homologous with other members of the PI3K family (**Fig. 1A**). Like the class I PI3Ks, VPS34 has a C-terminal helix that is essential for its catalytic activity (1), and this helix is intimately involved in a mechanism that suppresses basal activity of the catalytic subunit (2–5).

VPS34 uses phosphatidylinositol (PtdIns) as a substrate to produce PtdIns3P. The PtdIns3P recruits effectors bearing domains evolved to recognize the lipid headgroup. Among the common PtdIns3P-recognizing domains are the FYVE, PX, and PROPPINS. However, VPS34 produces PtdIns3P on many distinct compartments, with varying temporal changes in PtdIns3P concentration. For example, during amino-acid starvation that promotes autophagy, complex I activity is stimulated, whereas the majority of other VPS34 complexes are inhibited, because Unc-51-like autophagy activating kinase 1 (Ulk1) recruits autophagy-related gene (ATG)14L, enabling the kinase to phosphorylate Beclin 1 and enhance autophagy (6). This raises an important question as to how specific PtdIns3P-dependent pathways can be independently regulated. At least part of the answer to the question is that VPS34 never functions alone. It is likely that the yeast, Vps34, is not even stable on its own. While mammalian VPS34 appears to be well-behaved and stable even in the absence of any binding partners, it has minimal activity in this form. Although systematic comparison of the kinase activities among human VPS34 complexes has not been reported, human VPS34 activity is known to be increased by VPS15 (7). Similarly, kinase activities of yeast complexes I and II are higher than the Vps34/Vps15 heterodimer (2). These lines of evidence suggest that heterotetrameric assemblies are required for full VPS34 activities.

## VPS34 IS FOUND IN TWO PROMINENT FOUR-COMPONENT COMPLEXES

In mammalian cells, VPS34 forms two heterotetrameric core complexes known as complexes I and II. Complex I is composed of VPS34 (PIK3C3), VPS15 (p150, PIK3R4), Beclin 1, and ATG14L, whereas complex II has UV irradiation resistance-associated gene (UVRAG) instead of ATG14L (Fig. 1A). This difference, in only one subunit, dictates the specific localization of the activity of the two complexes. Although there are reports of Beclin 1, UVRAG, and ATG14L having roles in cells independent of the VPS34 subunit, we focus here only on their roles as components of VPS34-containing complexes. Autophagy is a catabolic recycling mechanism that degrades cytoplasmic constituents and organelles to regenerate amino acids, nucleotides, and lipids during starvation. Complex I is indispensable for the generation of PtdIns3P at the phagophore and thereby promotes autophagosome formation (8–10). In contrast, complex II regulates various intracellular events, including endocytic sorting (11), cytokinesis (12), autophagosome maturation (11), lysosome recycling (13), and LC3-associated phagocytosis (14).

Although complexes I and II are the best characterized, quantitative immunoprecipitation suggests that subcomplexes of complexes I and II also exist in cells (VPS34/VPS15 and VPS34/VPS15/Beclin 1) (15). Activities, stabilities, and compartmentalization of these complexes can be regulated. Although complexes I and II are activated under glucose starvation by AMP-activated protein kinase (AMPK)-mediated phosphorylation, there is no indication that this posttranslational modification (PTM) has an influence on assembly of the complexes. However, the Golgi-associated transmembrane protein, PAQR3, serves as a scaffold that promotes stabilization of complex I and Golgi compartmentalization under nonautophagic conditions (16). Upon glucose starvation, the PAQR3 scaffold is phosphorylated by AMPK, and complex I/PAQ3 locates to a punctate non-Golgi compartment. For yeast Vps34 complex II, the core complex can be reconstituted by association of two heterodimers: Vps30 (the yeast ortholog of Beclin 1) with Vps38 (the yeast ortholog of UVRAG) and Vps34 with Vps15 (2). However, it is not clear that this is the assembly pathway in cells. Both complexes I and II are stable complexes, so if there is an exchange between the Beclin 1/ATG14L and Beclin 1/UVRAG heterodimers, it is likely that this is a fairly slow process.

## CORE ARCHITECTURE OF COMPLEXES I AND II

**Fig. 2.**
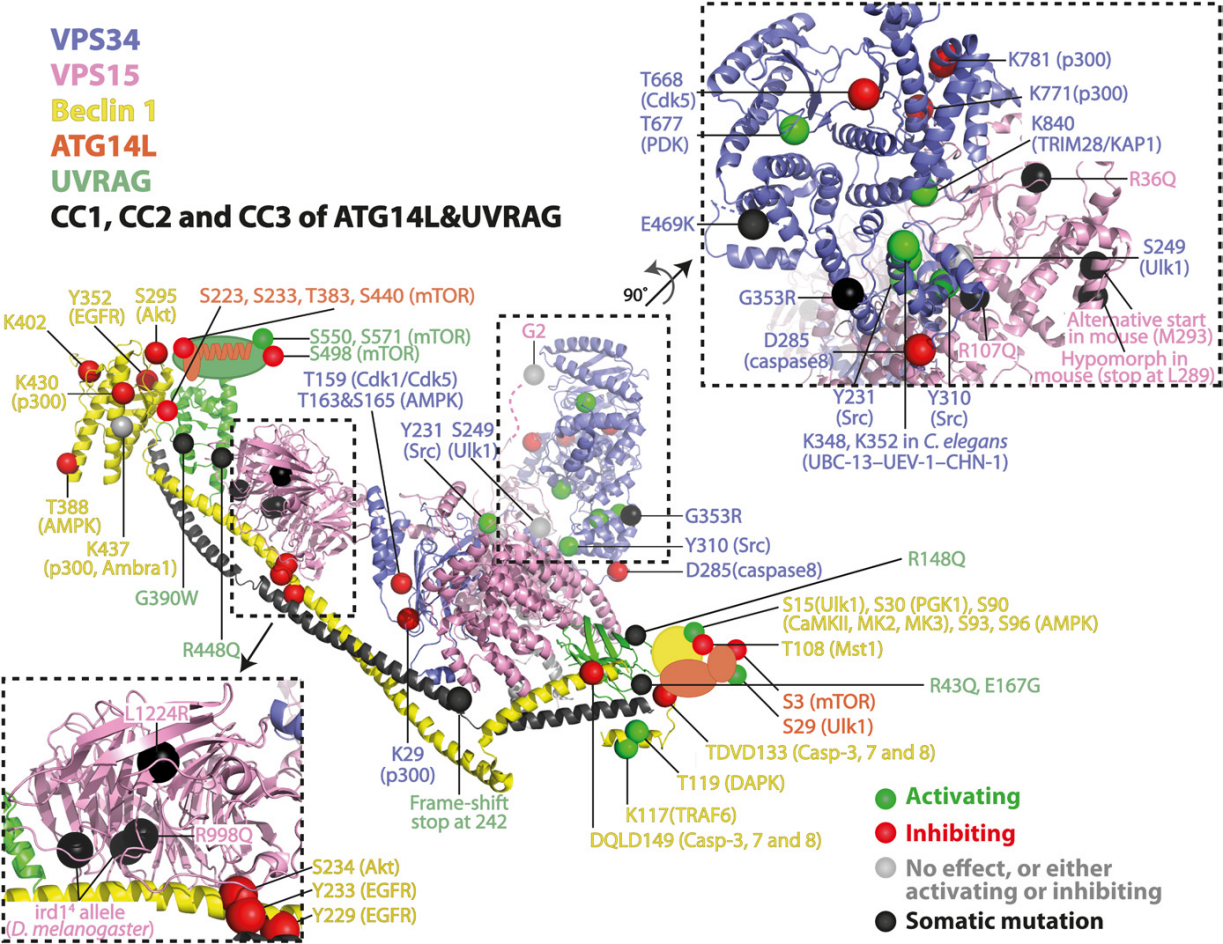
Overall views of human complexes I and II. PTMs and somatic mutations are mapped on the yeast complex II structure [Protein Data Bank (PDB) identification 5DFZ) because this is the highest resolution and most complete structure. Human numbering is used unless otherwise noted. Dark gray, CC1+CC2 in UVRAG and CC1+CC2+CC3 in ATG14L.

Complexes I and II are 1:1:1:1 heterotetramers, as shown by multi-angle light scattering, the X-ray crystal structure of complex II, and the cryo-EM structures of complexes I and II (2, 3, 17, 18). Structurally, both complexes I and II adopt a Y shape with VPS34/VPS15 forming a catalytic arm of the Y and Beclin 1/ATG14L (Vps30/Atg14 in yeast) or Beclin 1/UVRAG (Vps30/Vps38 in yeast) forming a regulatory arm (Fig. 1B and **Fig. 2**) (2, 3, 18). These two arms bind to membranes primarily via the aromatic finger motif in the BARA domain of Beclin 1 (see the Beclin 1 section) and the kinase domain of VPS34 (Fig. 1B). Yeast complexes I and II had equivalent activities on vesicles with high curvature. On flat membranes, yeast complex I showed no measurable activity, while complex II had robust activity (2). This preference of complex I for high curvature membranes might restrict the activity of complex I to membranes in the cell with high curvature, such as the omegasomes from which the isolation membrane emerges. There may be important differences between the yeast and human VPS34 complexes. X-ray crystallography and HDX-MS for the yeast complex II showed a stable association between the N-terminal pseudokinase domain of Vps15 and the C-terminal kinase domain of Vps34. The arrangement of the activation loop of VPS15 suggested that the crystallography had captured an inactive conformation in which a loop from VP15 interacts with the C-terminal helix from VPS34 to maintain the lipid kinase in an inactive state (2, 5). A study of the human complexes I and II by HDX-MS and electron microscopy suggested that the VPS34 kinase domain does not tightly associate with the VPS15 pseudokinase domain when the enzyme is active (5). A cryo-EM study of human complex I indicated that the VPS34 kinase domain is likely to take on an ensemble of orientations with respect to the rest of the enzyme (18). In other respects, the structural studies of the yeast and mammalian VPS34 complexes agree in the overall arrangement of the subunits. The greater mobility observed for the mammalian VPS34 kinase domain may be a unique property of the mammalian enzyme. The observation that yeast Vps34 cannot be expressed in the absence of Vps15 (2) may suggest that the yeast enzyme has a much closer association of Vps34 and Vps15 than is present in the mammalian enzymes. The structural work on the yeast Vps34 complex II was facilitated by a single-domain antibody construct that bound to the helical domain of the Vps34 subunit. It may be that this induced a more stable arrangement of the catalytic arm.

## ARCHITECTURE OF THE VPS34 SUBUNIT

The lipid kinase VPS34 subunit of complexes I and II consists of a C2 domain, a helical domain, and a kinase domain (Fig. 1A). The C2 domain is central to complexes I and II and forms key interactions with all three other core subunits (Figs. 1B, 2) (2, 3). This domain has a helical insertion (C2HH), with which it directly contacts the WD40 domain of VPS15 (Fig. 2) (2). A serine/threonine-rich loop in front of the C2HH is phosphorylated by Cdk1 and Cdk5 (T159) (19) or AMPK (T163) (15), which decreases the VPS34 activity. These modifications at the intersubunit-interface may weaken the complex stability. Caspase 8 cleaves VPS34 at D285, which is located at the junction between the C2 and the helical domain. Consistent with the structure, the resulting C-terminal fragment without the C2 domain shows decreased affinity for Beclin 1 and a reduction in VPS34 activity (20). In the helical domain, two highly conserved lysines (K348 and K352 in *Caenorhabditis elegans*) are poly-ubiquitinated by the UBC-13/UEV-1/CHN-1 complex. This stabilizes VPS34 and increases autophagosome maturation and clearance of cytoplasmic debris (21). Additionally, somatic mutations (G353R and E469K) were found in patients with desmoplastic melanoma, esophageal cancer, and metastatic melanoma (22–24). These mutated residues are not in the kinase domain and are not involved in binding any other subunit (Figs. 1A, 2); however, they may be important for membrane interaction or the conformational changes that accompany activation. Residue E469 is in a disordered loop of the human VPS34 and may be at the membrane-binding interface. A cryo-EM analysis of the orientations of complexes I and II on lipid monolayers suggested that what the authors referred to as the VPS34 C-terminal domain (which was actually a module consisting of most of the helical domain and the kinase domain) determines the orientation of the complex on lipid membranes, but contributes little to the affinity for membranes. Fully understanding this mutation will require a definitive analysis of the orientation of the enzyme on intact lipid bilayers. The G353 residue may be important for the flexibility of the C-terminal region of human VPS34 that has been noted in the cryo-EM study (18).

**Fig. 3.**
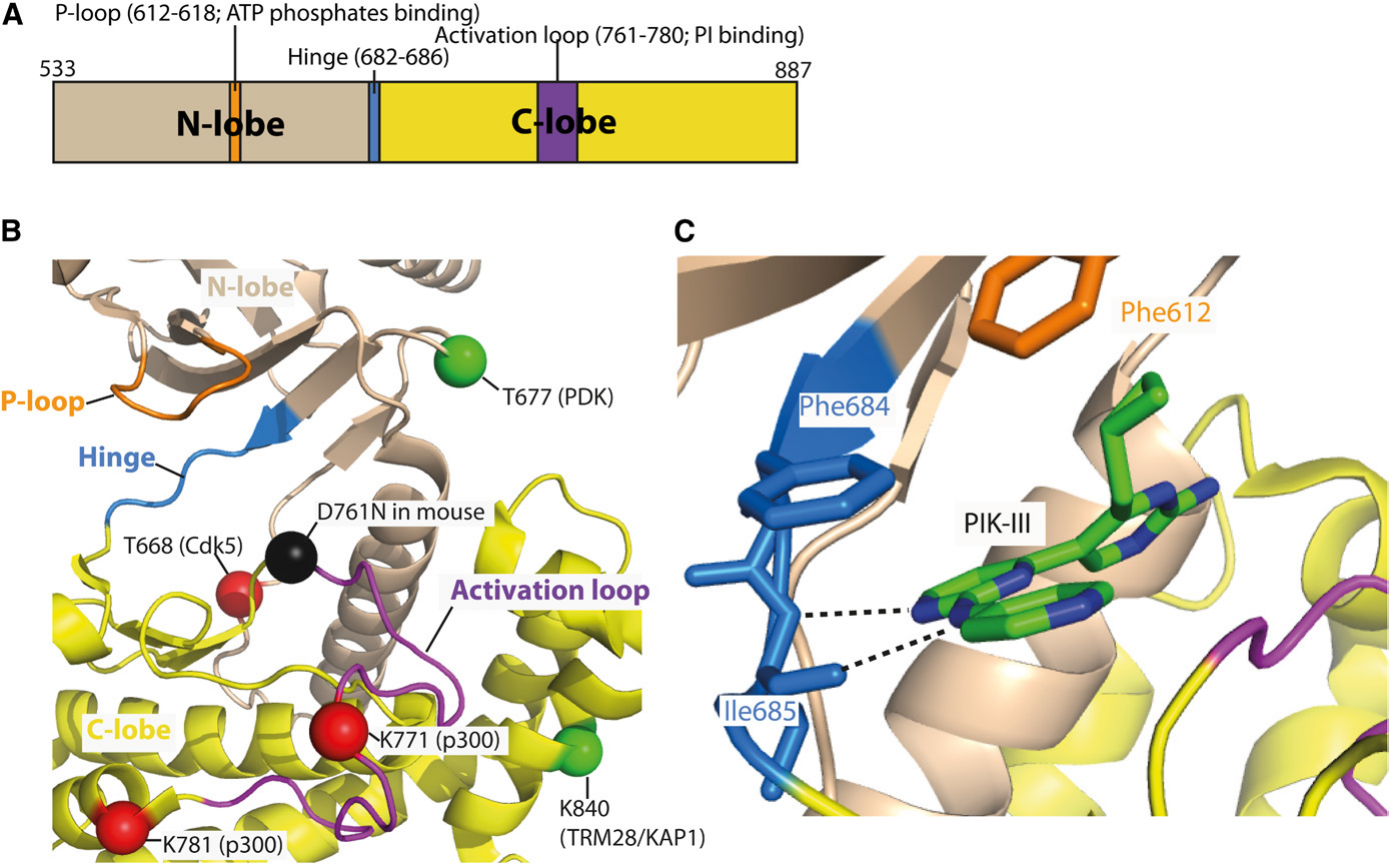
Close-up views of the kinase domain in human VPS34. A: A schematic representation of the kinase domain in human VPS34. B: A structural view of the ATP-binding pocket of human VPS34 (PDB identification 3IHY). PTMs are indicated in red for inhibiting and green for activating, respectively. A mouse knock-in mutation (D761N) is indicated in black. C: An example of VPS34-specific inhibitor, PIK-III, binding to the hinge in the ATP-binding pocket (PDB identification 4PH4).

The kinase domain undergoes various PTMs that affect the VPS34 activity (Fig. 1A and **Fig. 3B**). Among these, a recent study showed that p300 acetylates VPS34 at K29, K771, and K781 (Figs. 1A, 2) (25). Significantly, K771 is directly situated in the activation loop, which binds to the substrate PI (Fig. 3A, B). Thus, acetylation at K771 reduces the affinity of VPS34 for its substrate and thereby decreases VPS34 activity (25). Bilanges et al. (26) examined the importance of the kinase activity in mice by replacing a wild-type VPS34 gene (Pik3c3) with a gene fragment coding a kinase-dead version. This kinase-dead version has a point mutation, D761N, in the activation loop (Fig. 3B). A homozygous kinase dead knock-in mouse is embryonically lethal, showing that VPS34 activity is imperative for embryogenesis, organ function, and cell survival. A mutation in VPS34 helix α11 (H868R) meant to mimic the activating effect of the most common oncogenic mutant of PI3Kα (27) gave rise to a more active VPS34 and provided a tool to determine a mechanism whereby VPS34 could activate mTORC1 (Fig. 1A). Remarkably, heterozygous D761N/+ mice showed only mild autophagy defects in the liver and enhanced insulin sensitivity and glucose tolerance (26).The phenotype suggests that pharmacological inhibition could be well-tolerated and provide an alternative strategy to targeting type II diabetes.

## VPS34-SPECIFIC INHIBITORS

Autophagy can prevent tumor formation by removing superfluous or damaged proteins and organelles and thereby maintain cellular homeostasis (28). In contrast, during later stages of tumorigenesis, autophagy is used by cancer cells to survive metabolic and therapeutic stress. Chemical inhibition and downregulations of Atgs at this stage sensitize the cancer cells to various types of stress conditions (29, 30). Because complex I is involved in the early stage of autophagosome formation, VPS34 could be a useful drug target for cancer therapeutics. However, the classic VPS34 inhibitors, such as 3-methyladenine, wortmannin, and LY294402, also inhibit class I PI3Ks or PIKKs (31–35). Therefore, efforts have been made to develop more specific VPS34 inhibitors. Most members of this new generation of inhibitors that are VPS34 specific can be divided into two groups: bis-aminopyrimidine derivatives [VPS34-IN1 (36), PIK-III (37), and Compound 19 by Novartis (38)] and pyrimidinone derivatives by Sanofi [SAR405 (39), Compound 31 (40)]. In addition, SB02024, whose structure has not been disclosed, is also VPS34 specific (42). The active site of VPS34 is somewhat narrower than the class IA PI3Ks (1). These compounds target the hydrophobic region of the kinase domain ATP binding pocket (Fig. 3B, C). Like pan PI3K inhibitors, they bind the hinge between the N and C lobes of the kinase domain (Fig. 3C). Use of a morpholine group gave good selectivity for the PI3Ks, and substitutions in the group gave good selectivity for VPS34 (40). A recent study showed that SAR405 sensitized the urothelial carcinoma cell line and its cisplatin-resistant subline to cisplatin-induced cytotoxic effects (43), and inhibition by SB02024 increased sensitivity to sunitinib and erlotinib, suggesting that autophagy inhibition by VPS34-specific inhibitors could be an effective therapeutic strategy (**Table 1**). Because VPS34 is in both complex I and complex II, these VPS34 inhibitors inhibit both autophagy and endocytic pathways. The anti-autophagic strategy might be more effective and less toxic if this inherent off-target activity against complex II could be avoided. For this reason, it is important to understand the structural differences between the two complexes.

**TABLE 1.** Summary of VPS34-specific inhibitors

Compound Name	CAS Number	IC50 In Vitro (nM)	PDB Code	Reference
PIK-III	1383716-40-2	18	4PH4	(37)
VPS34-IN1	1383716-33-3	25	NA	(36)
Compound 19	1383716-46-8	15	5ENN	(38)
SAR405	1523406-39-4	1.2	4OYS	(39)
Compound 31	NA	2	4UWL	(40)
SB02024	NA	1	NA	(42)

CAS, Chemical Abstracts Service; NA, not available.

## VPS15: A PSEUDOKINASE THAT REGULATES VPS34

UVRAG-containing VPS34 complexes associate with the insulin receptor, and insulin stimulates VPS34 activity. A conditional deletion of Vps15 in mouse livers resulted in a positive influence on the metabolic effects in mouse models of obesity and type II diabetes (44). This has suggested that complex II and VPS15, specifically, might be targets for therapeutic intervention in diabetes. Although VPS15 has an N-terminal domain that has a kinase domain fold, it is thought to be a pseudokinase for the following reasons: First, except for its autophosphorylation in yeast (45), no substrate has ever been reported. Second, it lacks typical active-site motifs conserved among kinases, which include the GxGxxG motif in the ATP binding loop (P-loop), the HRD sequence in the catalytic loop, and the DFG sequence for magnesium ion binding in the activation segment (2). Third, in the yeast complex II crystal structure, the activation loop would prevent ATP binding, suggesting that the solved Vps15 structure is in an inactive conformation (2). VPS15 consists of a kinase domain (residues 24–315), a helical domain (residues 316–802), a structured linker region (residues 803–968), and a WD40 domain (residues 969–1358). In the fragment before the kinase domain, a glycine at residue 2 is conserved through evolution and is myristoylated to serve as a membrane anchor [Figs. 1A, 2; (46)].

The VPS15 N-terminal pseudokinase domain is important for VPS34 activity. MEFs expressing a Vps15 fragment lacking this region can initiate autophagy, but their autophagy flux is compromised. Furthermore, conditional knockout mice carrying the same truncated Vps15 fragment in skeletal muscles show severe muscle damage (47). The reason for these phenotypes is that this truncated form of VPS15 is unable to form complexes I and II. Conversely, the pseudokinase domain by itself is unstable in mice because a kinase domain fragment (1-289) derived from a nonsense mutation in a splicing site was not detected in Western blotting and caused hypomorphic mice with defects in the clearance of autophagic substrates, the induction of apoptosis, and neuronal migration (48). In addition, there are two cancer-related missense mutations in the pseudokinase domain. The R36Q mutation in the P-loop was found in colorectal cancer (46). A VPS15 R107Q mutation in close proximity to C2/helical linker helix in VPS34 was found in metastatic melanoma patients and could affect the orientation of the VPS34/VPS15 assembly (Fig. 2) (49). The C-terminal WD40 domain of VPS15 is essential for interaction with GTP-RAB5 on early endosomes (50, 51). Several somatic missense mutations in humans and *Drosophila melanogaster* have been found in the WD40 domain (Figs. 1A, 2). A ciliopathy mutation (R998Q) (52) and a neurodevelopmental disease mutation (L1224R) (48) were found in humans. Furthermore, an immune response-deficient mutant (ird1) allele ird1^4^, which is susceptible to *Escherichia coli* and *Micrococcus luteus* bacterial infection, was found in *D. melanogaster* (G986D and V1337I) (53). These mutations may cause the instability of the WD40 domain, which may in turn destabilize the VPS34 complexes (48).

## BECLIN 1: A MEMBRANE ADAPTOR REGULATED BY PTMs

The Beclin 1 gene (*BECN1*) was originally found in a transcription mapping study of the BRCA1 locus (54). Subsequently, the high similarity of Beclin 1 to the product of the fundamental yeast autophagy gene, *ATG6*/*VPS30*, was recognized, and, therefore, it was the first-characterized mammalian autophagy gene (55). Beclin 1 has also attracted attention as a haploinsufficient tumor suppressor gene, as it was found to be monoallelically deleted in several cancers (56–58). However, Laddha et al. (59) have recently proposed that Beclin 1 was incorrectly reported to be a tumor suppressor because of its proximity to the BRCA1 gene, as deletions were found to contain either both BRAC1 and Beclin 1 or BRAC1 alone, indicating that BRCA1 is the driver of tumorigenesis. Beclin 1 contains four domains of known structure: a BH3 domain (residues 105–125), a short coiled-coil domain 1 (CC1) (residues 139–171), a longer coiled-coil domain 2 (CC2) (residues 171–269), and a BARA domain (residues 275–449). Beclin 1 has numerous PTMs that mediate its localization, binding partners, and stability. When the known PTMs are mapped on the structure, it can be seen that autophagy-promoting modifications are largely found in the N terminus and BH3 domain subunits of complexes I and II are shown in **Table 2**. In contrast, autophagy-inhibiting PTMs are primarily found in the CCDs and the BARA domain (Fig. 1A). For example, Beclin 1 is phosphorylated in its N-terminal domain at S15 by ULK1 and at S93/S96 by the AMPK in complexes I and II. Both PTMs activate the VPS34 complexes (6, 15, 60). From a structural perspective, it is not clear how these phosphorylations lead to an activation. BH3 domain-containing proteins belong to a family of apoptosis regulators, but Beclin 1 does not have any apoptotic potential. Nevertheless, the apoptotic protein, Bcl-2, can bind Beclin 1 and reportedly sequesters it to reduce autophagy (61). However, some studies have not identified Bcl-2 as a binding partner of the VPS34 complexes (10, 62), although Liang et al. (63) could purify a complex containing VPS34, VPS15, Beclin 1, and UVRAG using a viral homolog of Bcl-2 (vBcl-2). This suggests that vBcl-2 does not dissociate human complex II. Interestingly, Beclin 1 is phosphorylated in its BH3 domain on T119 by death-associated protein kinase (DAPK), which in turn promotes the segregation of Bcl-2 and Beclin 1 (Figs. 1A, 2) (64). Furthermore, Young et al. (41) discovered that the BH3 domain is highly protected from hydrogen-deuterium exchange of human complex I in the presence of NRBF2 and, in turn, activates the VPS34 complex I in vitro. It remains to be determined how the N terminus and BH3 domain contribute to VPS34 activity. In the CC2 of Beclin 1, three intriguing phosphorylation sites can be found. S229 and S233 are phosphorylated by epidermal growth factor receptor (EGFR) tyrosine kinase and S234 is phosphorylated by Akt (65, 66). All three phosphorylation sites are in direct proximity to the VPS15 WD40 domain and could consequently impair the assembly of the heterotetrameric complexes and thus reduce kinase activity (Fig. 2). The BARA domain of Beclin 1 is a stretch of ∼200 amino acids, which folds into a globular fold comprised of three β-sheet-α-helix repeats (67, 68). It shows a strong binding to lipid membranes, with a principal component of the binding contributed by a surface loop with three consecutive aromatic amino acids, Phe359, Phe360, and Trp361, at its tip (the aromatic finger motif) (68). Mutating this motif decreased the membrane binding in vitro, and mutating three analogous residues in yeast Vps30 leaves complex II completely inactive and unable to bind to liposomes (2, 68). Several PTMs can be found in the BARA domain, which might either affect the proper fold of the domain or membrane binding. Especially notable is the phosphorylation at S295 by Akt, a site that is directly at the membrane interface (Fig. 2) (66).

**TABLE 2.** PTMs in class III PI3K subunits

Subunit	Position	Type	Enzyme	Region	Reference	Effect	Position in Yeast
VPS34	K29	Acet.	p300	C2	(25)	Inhibits VPS34-Beclin 1 association, enhances Rubicon interaction	H28
	T159	Phos.	Cdk1/Cdk5	C2HH	(19)	Inhibits interaction with Beclin 1	NA
	T163, S165	Phos.	AMPK	C2HH	(15)	Inhibits autophagic complex assembly	NA
	S249	Phos.	Ulk1	C2	(60)	No effect	G237
	D285	Caspase-mediated cleavage	Caspase 8	C2/helical linker	(20)	Abolishes kinase activity, decreases interaction with Beclin 1	Q296
	T668	Phos.	Cdk5	Kinase (N-lobe)	(19)	Inhibits lipid kinase activity	T656
	T677	Phos.	PDK	Kinase (N-lobe)	(105)	Activates autophagy	P665
	K771	Acet.	p300	Kinase (C-lobe)	(25)	Disrupts VPS34-PtdIns interaction	K759
	K781	Acet.		Kinase (C-lobe)	(25)	K781Q mutation attenuates VPS34-PtdIns interaction	P769
	K840	SUMO.	TRIM28/KAP1	Kinase (C-lobe)	(106)	Enhances association with Beclin 1	L828
	Y231	Phos.	Src	C2/helical linker	(107)	Stimulates VPS34 translocation to the plasma membrane induced by insulin, and activation there	E219
	Y310	Phos.		Helical	(107)		A321
	k348, k352 (*C. elegans*)	K63-poly-polyubiquitylation	UBC-13–UEV-1–CHN-1	Helical	(21)	Stabilizes VPS-34 (*C. elegans*)	K339 K343
VPS15	2G	Myristoyl.	?	N terminus	(46)	G2A single mutant is similar to WT, phenotypes are enhanced when G2A is combined with one C-terminal deletions (*S. cerevisiae*)	2G
Beclin 1	S15	Phos.	ULK1	IDR	(6)	Enhances activity of complex I	NA
	S30	Phos.	Acetylated PGK1	IDR	(108)	Enhances the ability of VPS34 to bind to PtdIns thereby increasing complex I activity	S15
	S90	Phos.	CaMKII	IDR	(109)	Promotes activation of autophagy via Beclin 1 dissociation from Bcl-2	D78
	S90	Phos.	MK2 and MK3	IDR	(110)	Promotes autophagy	D78
	S93, S96	Phos.	AMPK	IDR	(15)	Activates the pro-autophagy Vps34 complex, and induces autophagy	L81, S85
	S90,93	Phos.	?	IDR	(111)	Critical for maximally efficient autophagy	D78, L81
	T108	Phos.	Mst1	BH3	(112)	Inhibits the activity of complex I and suppresses autophagy	S154
	K117	K63-linked ubiquitination	TRAF6	BH3	(113)	Critical for TLR4-triggered autophagy in macrophages	N162
	T119	Phos.	DAPK	BH3	(114)	Promotes the dissociation of Beclin 1 from Bcl-XL and the induction of autophagy	M164
	TDVD^133^ and DQLD^149^	Caspase-mediated cleavage	Casp-3, 7 and 8	CC1	(115)	Yields fragmentation of Beclin 1, which lacks the autophagy-inducing capacity	
	Y229, Y233 and/or Y352	Phos.	EGFR	CC2 and/or BARA	(65)	Decreases Beclin 1-associated VPS34 kinase activity	K282, Q286 and/or Y419
	S234, S295	Phos.	Akt	CC2 (and possibly BARA)	(66)	Inhibits autophagy and promotes the formation of the Beclin 1/14-3-3/vimentin intermediate filament complex	N287 (and possibly E348)
	T388	Phos.	AMPK	BARA	(116)	Causes a higher affinity for BCL2	S459
	K402	K48-linked ubiquitination	?	BARA	(117)	Causes proteasome-mediated degradation, de-ubiquitinated by ataxin3	K498
	K430, K437	Acet.	p300	BARA	(118)	Inhibits autophagosome maturation and endocytic trafficking by promoting the recruitment of Rubicon.	K520, K527
	K437	K63-linked ubiquitination	Ambra1	BARA	(119)	Enhances the association with VPS34 to promote Vps34 activity	K527
ATG14L	S3	Phos.	mTOR	N-ter to CXXC	(72)	Inhibits complex I activity	NA
	S223	Phos.		C-ter			NA
	S233	Phos.		C-ter			NA
	T383	Phos.		C-ter			V288
	S440	Phos.		BATS			NA
	R423, R442	—	—	BATS	(75)	PtdIns(4,5)P2 binding, important for binding to the autophagosome	NA
	S29	Phos.	Ulk1	N-terminal before CXXC	(70)	Important for complex I activity	NA
UVRAG	S493	Phos.	mTOR	C-Ter	(89)	Not known	NA
	S498	Phos.			(13, 89, 120, 121)	Incrcases the association with Rubicon inhibits VPS34, decreased endosome maturation	NA
	S508	Phos.			(89)	Not known	NA
	S518	Phos.			(13, 120)	Not known	NA
	S522	Phos.			(89)	Not known	NA
	S549	Phos.			(89, 120)	Not known	NA
	S550	Phos.			(13, 89, 120)	Increases VPS34 complex II activity and promotes autophagosome-lysosome reformation	NA
	S571	Phos.			(13, 120)	Increases VPS34 complex II activity and promotes autophagosome-lysosome reformation	NA
	S582	Phos.			(89)	Not known	NA
	S689	Phos.			(13, 120)	Not known	NA

## ARCHITECTURE AND FUNCTION OF THE AUTOPHAGY-SPECIFIC ATG14L SUBUNIT

ATG14L is the defining component for VPS34 complex I. It consists of an N-terminal domain (residues 1–72), a short coiled-coil (CC1) (residues 73–107), two longer coiled-coils (CC2 and CC3) (CC2 residues 108–160, CC3 residues 161–206), a C-terminal domain (residues 233–412), and a Barkor/Atg14L autophagosome targeting sequence (BATS) domain (residues 412–493). The N-terminal part of ATG14L contains a pair of CXXC motifs (C, cysteine; X, any amino acid) that are the most conserved regions through evolution (CXXC1 residues 44–47, CXXC2 residues 56–58). These regions are important for ATG14L localization to the ER (69). For complex I to localize to the autophagosome and to be activated, the upstream kinase Ulk1 complex is required. The serine/threonine kinase, Ulk1, together with its associating proteins, ATG13 and FIP200, phosphorylate ATG14L at S29. This phosphorylation is stimulated by amino acid starvation, mTOR inhibition, and glucose deprivation and is important for the activation of complex I and autophagosome formation (70). The S29 phosphorylation and complex I activity are decreased in the context of a Huntington’s disease model mice (71). Residue S29 is located in an extension before the CXXC motif, which exists only in metazoans. This N-terminal region is distant from the kinase domain of VPS34 and the putative membrane-interacting region (Fig. 2); therefore, it is not clear how this phosphorylation upregulates the activity of complex I.

Complex I activity is inhibited by multiple phosphorylations on ATG14L in its C-terminal domain by mTOR (Fig. 1A, B) (72). ATG14L possesses an extended C terminus called the BATS domain, which is unique to mammals (Fig. 1A, B). The BATS domain confers on complex I the ability to bind to PtdIns-containing vesicles much more readily than complex II in vitro, and in cells it enables complex I to localize to the ER (18). At the C-terminal end of the BATS domain, there is an α-helix with similarity to the ArfGAP1/amphipathic lipid packing sensor (ALPS) motif, whose hydrophobic residues are known to be inserted into membranes (73). It is an amphipathic helix enriched in serine and threonine on its polar side and has three essential bulky hydrophobic residues (tryptophan, phenylalanine, and tyrosine) at its apolar side. This helix is crucial for the localization of ATG14L to the autophagosome in vivo and for membrane association in vitro (74). Mutations of the three hydrophobic residues to arginine (W484R, F485R, and Y488R) are enough to disturb the localization of ATG14L to the phagophore (74). Also, the R423 and R442 residues, which are in the BATS domain but outside of the ALPS helix, are known to be important for PtdIns(4,5)P2 binding (75). A high resolution structure of complex I will be required to understand the ATG14L-mediated activation/inhibition mechanism.

## ARCHITECTURE AND FUNCTION OF THE ENDOCYTIC SORTING-SPECIFIC UVRAG SUBUNIT

UVRAG is the fourth subunit of the VPS34 complex II. The UVRAG gene was first identified in a genetic screen in 1997 in which it was shown to partially rescue UV sensitivity in xeroderma pigmentosum cells (76). Xeroderma pigmentosum is a genetic condition in which the DNA repair mechanisms for UV light are impaired. The human UVRAG gene is located on chromosome 11q13, which is a chromosomal region that is closely correlated to organ rotation/heterotaxy syndromes (77, 78) and human cancers, such as colon, breast, and gastric cancer (76). Furthermore, similar to Beclin 1, UVRAG is also thought to have tumor suppressor activity, as it is regularly monoallelically deleted or mutated in these cancers (63, 79–81). UVRAG was found to localize to Rab9- and Rab5-positive endosomes as part of VPS34 complex II, and it was thereafter shown that UVRAG is not involved in the initiation of autophagy but functions mainly in endocytic trafficking and potentially in autophagosome maturation and autophagosome-lysosome fusion (82, 83). Although deletion analysis has suggested that the C-terminal WD40 domain of VPS15 interacts with GTP-Rab5 on early endosomes (51), this would not account for colocalization of only UVRAG-containing complexes on Rab5-positive endosomes.

UVRAG consists of five distinct regions: a proline-rich domain (residues 1–52), a lipid-binding C2 domain (residues 53–164), a short CC1 (residues 195–226), a longer CC2 (residues 240–333), a BARA2 domain (residues 364–464), and a C-terminal domain (residues 464–699). The C2 domain was shown to bind to PtdIns3P, PtdIns4P, and PtdIns5P with residues K78 and R82, although the C2 domain is distant from the putative membrane plane facing the VPS15 helical domain (Figs. 1B, 2) (84). Interestingly, three somatic mutations were found either in the C2 domain [R148Q (81)] or in the region between C2 and CC1 [R43Q (85), E167G (86)]. The BARA2 domain has a similar fold to the Beclin 1 BARA domains, as it folds into the globular domain of one β-sheet and two α-helix repeats. However, no direct membrane binding has been detected so far (2). Two somatic mutations were reported in the BARA2 domain at G390W (87) and R448Q (88) in colorectal and bladder cancer, but they are not located at the membrane interface. Interestingly, human HCT116 colon cancer cells contain a dominant monoallelic deletion of one or two adenines in a cluster of 10 adenine nucleotides. This generates a premature stop codon at the junction of CC1 and CC2 (Figs. 1A, 2) (63, 79). The truncated fragment of UVRAG consisting only of the proline-rich C2 and the CC1 domain causes defective autophagy and increased tumorigenesis. We have previously shown that the corresponding fragment in yeast, Vps38 (UVRAG ortholog), is able to form a stable heterodimer with the NTD-CC1 fragment of Vps30 (Beclin 1 ortholog) (2). Hence, the frameshift truncated UVRAG may be able to sequester Beclin 1 away from the VPS34 complexes in vivo, thereby impairing autophagy. In fact, the UVRAG frameshift fragment can also bind the wild-type full-length UVRAG and thereby reduces the available full-length UVRAG for other cellular pathways (79).

The C-terminal region of UVRAG is considered to be unstructured and significantly longer than the C terminus of Vps38 (UVRAG ortholog). Sequence alignment showed that the amino acid stretch of 465 to 699 is unique to its mammalian counterpart (2). Intriguingly, numerous phosphorylation sites were identified in this region (12, 79, 83). As the C terminus is directly at the membrane interface (Figs. 1B, 2), it is tempting to speculate that phosphorylations would alter membrane binding and, thereby, VPS34 activity. Two different groups have characterized phosphorylation sites by mTORC1. Kim et al. (89) showed that UVRAG is phosphorylated at S498 under nutrient-rich conditions by mTORC1, which increases the association with Rubicon. Consequently, VPS34 activity is decreased and endosome and autophagosome maturation is inhibited (89). In contrast, Munson et al. (13) discovered that S550 and S571 are phosphorylated by mTORC1 under amino acid-rich conditions. These phosphorylations cause an activation of VPS34 complex II. Mutating these residues leads to a decrease of PtdIns3P at the lysosome and an increase of lysosomal tubules that are needed for the reformation of lysosomes out of the autophagosome, called autophagosome-lysosome reformation (13).

## ACCESSORY SUBUNITS ASSOCIATED WITH COMPLEXES I AND II

In addition to the core subunits of complexes I and II, accessory subunits have been characterized that control the activities and localization of the complexes. Four such components that have been extensively characterized are the proteins, NRBF2, Rubicon, PAQR3, and AMBRA1. NRBF2 and Rubicon associate to form stable associations with complexes I and II, respectively. In contrast, AMBRA1 only weakly associates with complexes I and II (90).

NRBF2 is a fifth component of human complex I, although there is disagreement as to the nature of its regulation of kinase activity (91–93). Structurally, NRBF2 has an N-terminal microtubule-interacting and targeting (MIT) domain and a coiled-coil dimerization domain at the C terminus. The MIT and the coiled-coil domains are flanked by an intrinsically disordered region (IDR). The MIT domain is responsible for the binding to complex I (17, 41, 92, 93). Both human NRBF2 and its yeast homolog, Atg38, use their N-terminal MIT domain to interact with the N termini of Beclin 1 (Vps30) and ATG14L (Atg14) (17, 41). The coiled-coil domain of NRBF2 and its yeast ortholog, Atg38, are known to homodimerize (17, 41, 94). NRBF2 forms a stable complex with complex I, and the stoichiometry between complex I and NRBF2 can be 1:1 (a homodimer of a heteropentameric complex I+NRBF2) or 1:2 (one copy of complex I bound to one NRBF2 homodimer), depending on the concentration of NRBF2 (17). The IDR is phosphorylated by mTOR at S113 and S120. This decreases binding of NRBF2 to complex I subunits, which in turn decreases VPS34 activity (95). This mechanism helps to inhibit complex I when amino acids are replete and mTORC1 is active. NRBF2-deficient mice show focal liver necrosis and ductular reaction (92). In Alzheimer’s disease cell models, NRBF2 is involved in the downregulation of the amyloid β precursor protein and its C-terminal fragments (96). Although both NRBF2 and PAQR3 interact with complex I and increase its activity, they coordinately regulate complex I, with the NRBF2 binding to the N-terminal ends of the coiled-coil regions of the Beclin 1 and ATG14L (17, 41), while PAQR3 interacts with the N terminus of Beclin 1, the C-terminal half of ATG14L, and the pseudokinase and WD40 domains of VPS15 (16). PAQR3 not only increases the complex I activity but also regulates the compartmentalization of complex I.

Complex I localizes to the phagophore/isolation membrane, autophagosome, and ER, and can associate with AMBRA1. AMBRA1 is a 1,300 residue protein that has an N-terminal WD40 domain and a vast region that is thought to be intrinsically disordered (97). AMBRA1 acts as a hub coordinating several processes to promote autophagy and regulate mTOR signaling. It interacts with Beclin 1 and increases VPS34 activity (98). However, unlike NRBF2, it appears that AMBRA1 forms only transient interaction with either complex I or complex II (90).

While complex I upregulates autophagy at an early step, a UVRAG-containing complex (presumably complex II) forms a stable interaction with Rubicon through an interaction with Beclin 1, localizes on late endosomes/lysosomes, and negatively regulates later events in both autophagy and the endocytic pathway (10, 62, 99). However, the role of Rubicon is not simple because it also has been reported to have a positive influence on complex II activity in a different context. Rubicon is required for the noncanonical phagocytosis known as LC3-associated phagocytosis (14). Rubicon interacts with Rab7-GTP through a C-terminal FYVE-like domain known as the Rubicon-homology domain (100). A Rubicon-homology domain is found in two other proteins related to Rubicon, PLEKHM1 (101) and Pacer (102). Although Rubicon and UVRAG exist in the same complex, reports differ as to the effect of Rab7 binding. Q. Zhong and colleagues reported that Rab7 and Rubicon exist in the same complex, but they could see no interaction between Rab7 and UVRAG by immunoprecipitation (100). This study also showed direct competition between Rab7 and UVRAG for binding to Rubicon in vitro and in cells. While T. Yoshimori and colleagues did not describe such a competition between Rab7 and UVRAG binding to Rubicon, their immunoprecipitation analysis indicates that Rab7 binds to complex II via Rubicon (101). PLEKHM1 has a domain homologous with the Rab7-interacting domain of Rubicon, and like Rubicon, PLEKHM1 inhibits endocytic sorting (101). Unlike Rubicon, PLEKHM1 does not interact with complex I. Rubicon binding is antagonized by Pacer and it enhances autophagosome maturation (102). Pacer, like Rubicon, interacts with complex II in a manner that requires Beclin 1. UVRAG is phosphorylated by mTOR (see the UVRAG section), leading to Rubicon binding, while dephosphorylation of UVRAG causes dissociation of the UVRAG-Rubicon interaction, enabling UVRAG to associate with the HOPS complex, which is involved in late endosome-lysosome fusion (89). The HOPS complex is also known to bind to STX17. However this interaction is mutually exclusive with the HOPS-UVRAG interaction (103). STX17 is also known to bind to complex I, and the interaction is enhanced at the ER/mitochondria contact site upon amino acid starvation by an unknown mechanism (104). At least in the case of complex II, associating proteins might not bind simultaneously; rather, each of them may bind as part of a cascade of interactions in the pathway.

## CONCLUDING REMARKS

The VPS34 complexes are activated in unique contexts and the mechanisms of their spatiotemporal regulation are now emerging. Structures of the complexes are beginning to clarify the organization of the complexes and the accessory subunits with which they associate. This has begun to offer interpretations to the sometimes bewildering range of interactions that have been reported for these complexes. The PTMs of the VPS34 complex subunits have a wide range of complex-specific influences, and suggest that it may be possible to devise approaches to inhibit specific VPS34 pathways.
